# Screening prior to Breast Cancer Diagnosis: The More Things Change, the More They Stay the Same

**DOI:** 10.1155/2013/327567

**Published:** 2013-09-18

**Authors:** Erica B. Friedman, Jennifer Chun, Freya Schnabel, Shira Schwartz, Sidney Law, Jessica Billig, Erin Ivanoff, Linda Moy, Deborah Axelrod, Amber Guth

**Affiliations:** ^1^Department of Surgery, NYU Langone Medical Center, 160 East 34th Street, 4th Floor, 550 First Avenue, New York, NY 10016, USA; ^2^School of Medicine, NYU Langone Medical Center, 160 East 34th Street, 4th Floor, 550 First Avenue, New York, NY 10016, USA; ^3^Department of Radiology, NYU Langone Medical Center, 160 East 34th Street, 4th Floor, 550 First Avenue, New York, NY 10016, USA

## Abstract

*Purpose*. In November 2009, the U.S. Preventative Service Task Force (USPSTF) revised their breast cancer screening guidelines. We evaluated the pattern of screening subsequent to the altered guidelines in a cohort of women. *Methods*. Our database was queried for the following variables: age, race, method of diagnosis, mass palpability, screening frequency, histology, and stage. Statistical analyses were performed using Pearson's chi-square and Fisher's exact tests. *Results*. 1112 women were diagnosed with breast cancer from January 2010 to 2012. The median age at diagnosis was 60 years. Most cancers were detected on mammography (61%). The majority of patients had invasive ductal carcinoma (59%), stage 0 (23%), and stage 1 (50%) cancers. The frequency of screening did not change significantly over time (*P* = 0.30). However, nonregular screeners had an increased risk of being diagnosed with later stage breast cancer (*P* < 0.001) and were more likely to present with a palpable mass compared to regular screeners (56% versus 21%; *P* < 0.001). *Conclusions*. In our study, screening behavior did not significantly change in the years following the USPSTF guidelines. These results suggest that women who are not screened annually are at increased risk of a delay in breast cancer diagnosis, which may impact treatment options and outcomes.

## 1. Introduction

Breast cancer is the most common invasive cancer in women, with upwards of 1 in 8 women being affected during their lifetime. In 2013, it is estimated that over 230,000 women will be diagnosed with breast cancer. While the incidence rate for invasive breast cancer has increased slightly from 2005 to 2009, the death rate continues to steadily decline [1]. The consistent reduction in breast cancer mortality began in the 1990s, around the time medicare approved coverage for screening mammography [2], and is largely a reflection of improvements in early detection and/or treatment [3].

Despite years of clinical research that demonstrate a reduction in breast cancer mortality attributable to screening mammography [3–6], in November 2009, the United States Preventative Services Task Force (USPSTF) published updated guidelines for breast cancer screening that markedly differed from their last update in 2002 and significantly decreased recommended screening. These updates recommended against routine mammographic screening for women aged 40–49 suggested biennial rather than annual screening for women aged 50 to 74 and opposed screening for women aged 75 and older. These revised guidelines led to a great deal of controversy as they are divergent from those of the American Cancer Society (ACS) and the National Comprehensive Cancer Network (NCCN) that call for annual screening mammography starting at age 40 for asymptomatic women and continuing for as long as a woman is in good health [7]. 

Disparate recommendations from professional associations can be the source of confusion among the public and may affect screening behaviors. The purpose of this study was to evaluate the pattern of screening subsequent to the revised USPSTF guidelines in a population of women who were newly diagnosed with breast cancer at our institution.

## 2. Methods

The Breast Cancer Database (BCD) at New York University Langone Medical Center (NYULMC) is a prospective database established in January 2010 and intended to include all individuals undergoing definitive breast cancer surgery at our institution. The database includes elements of prediagnosis personal and family history, screening history, method of diagnosis, stage at diagnosis, histopathological characteristics of the tumor, details of treatment, and outcomes. The BCD was queried to identify all women ≥40 years of age presenting for definitive surgical management of intraductal carcinoma and early or locally advanced breast cancer. We queried the database for the following clinical and pathological variables: age, race, how the cancer was first detected, mass palpability, screening frequency, stage, histology, and ER/PR/Her2-neu status.

Patients were divided into 3 age groups based on USPSTF guidelines: (1) women aged 40–49, who would be excluded from recommendations for screening, (2) women aged 50–74, who would be recommended for biennial screening, and (3) women aged 75 years and older, who would be excluded from screening. Screening frequency was defined as annual (regular), biennial, or nonregular and was assessed by questionnaire data and confirmed by medical chart review when available. Statistical analyses were performed using SAS version 9.3 (SAS Institute, Cary, NC). Variables of interest, including clinical and histopathological characteristics, were compared across the screening years (January 2010 through December 2012) using Pearson's chi-square and Fisher's exact tests. This study was approved by the Institutional Review Board of NYULMC.

## 3. Results

There were a total of 1112 women with a new diagnosis of ductal carcinoma in situ or invasive breast cancer presented to NYU for definitive surgical management. The median age at diagnosis was 60 years and ranged from 40 to 95 years. In this cohort, 270 (24%) patients were between the ages of 40 to 49 years at the time of their breast cancer diagnosis, 696 (63%) patients were between the ages of 50 to 74 years, and the remaining 146 (13%) patients were 75 years of age and older. The majority of patients were Caucasian (76%) and had no family history of breast cancer in a first degree relative (74%) and no personal history of prior breast cancer (89%).

Overall, mammography was the modality of cancer detection in 684 (61%) women and remained the first method of detection in the majority of women over the study time period without significant change (*P* = 0.40). Less than a third of patients presented with a palpable mass (29%), while the remaining 787 (71%) women presented with clinically occult tumors. There was no statistically significant difference in tumor palpability in our patient population over time (*P* = 0.31). The majority of women (69%) were annual (regular) screeners, and the frequency of screening (annual versus biennial versus not regular) did not change significantly over time (*P* = 0.30) (Table 1).

The majority of our patients presented with stage 0 (23%) and stage 1 (50%) breast cancers that were ER positive (82%), PR positive (69%), and Her2-neu negative (66%). The most common histology was invasive ductal carcinoma (59%). These tumor characteristics did not change significantly over time: stage (*P* = 0.66), ER (*P* = 0.12), PR (*P* = 0.29), and histology (*P* = 0.79) (Table 1). 

While the majority of women in each age group were annual screeners, the proportion of annual, biennial, and nonregular screeners did vary significantly between age groups (*P* < 0.0001). Specifically, there were significantly more nonregular screeners among women aged 40–49 years (28%) compared to women aged 50–74 years (15%) and women aged 75 years and older (18%) (Table 2). The screening behaviors of women between the ages of 40 to 49 years and women aged 75 years and older did not change significantly from 2010 to 2012. However, there was a significant change in the screening behavior of women aged 50–74 (*P* = 0.02), with a slight decline in the proportion of annual screeners and a small increase in the proportion on nonregular screeners.

When we looked at screening frequency, breast cancer stage, and tumor palpability, we found that women who were regular (annual) screeners were most likely to present with early breast cancer, including carcinoma in situ (27%) or stage I (53%) (Table 3). These women were also most likely to present with nonpalpable cancers (79%) (Table 4). Conversely, women who were not regular screeners had an increased risk of being diagnosed with later stage breast cancer (*P* < 0.0001) and were more likely to present with a palpable mass when compared to women who were regular screeners (56% versus 21%; *P* < 0.0001).

## 4. Discussion 

Over the last 25 years, screening mammography, along with improvements in therapy, is responsible for a substantial decrease in breast cancer mortality [5]. Multiple reports from randomized controlled trials show improved survival in women who undergo screening mammography compared to those who do not as well as an independent association between screening detection and improved disease-specific and overall survival [8]. Specifically, a 2009 meta-analysis of these trials found a statistically significant reduction in breast cancer mortality for women in 2 age groups, aged 39–49 (15%) and aged 50–59 (14%), randomly assigned to screening mammography versus those who were not assigned to screening (controls) [9]. 

Despite the similar mortality reductions between the two age groups, the 2009 USPSTF updated guidelines largely deviated from prior guidelines that recommended annual mammographic screening beginning at age 40. The update recommended against routine mammographic screening for women aged 40–49. The rationale for this change was that, given the lower incidence of breast cancer in this age group, the harms of screening, including high rates of false positives and need for additional imaging, outweighed the potential benefits of early detection [9]. Additionally, the 2009 guidelines advocate for biennial rather than annual screening for women aged 50 to 74 a recommendation based on statistical modeling that suggested the longer interval between screening would decrease false positives while maintaining the mortality benefit of annual screening [10]. Lastly, the USPSTF felt that current evidence was insufficient to support screening mammography in women aged 75 and older. The goal of this study was to evaluate the screening behaviors of women newly diagnosed with breast cancer at our institution in the time period following the updated guidelines.

In the three years since the publication of the updated USPSTF guidelines, we found that the frequency of screening in our population did not significantly change. During this period, the majority of women in each age group were annual screeners. Perhaps not surprisingly, the greatest proportion of nonscreeners was found in women aged 40–49, where the current USPSTF guidelines are in the starkest contrast to guidelines from other agencies. The breakdown of screening behavior in this age group did not change significantly over the study time period.

Interestingly, we did find a significant change in the screening behavior of women aged 50–74. While there was a decline in the proportion of annual screeners, this was not matched by an increase in biennial screening, as one might expect based on the updated guidelines. Instead, we found a small increase in the proportion of nonregular screeners. Though it is impossible to prove the impetus behind the change in screening behavior in this age group, we can speculate that an underlying cause may be confusion stemming from disparate screening recommendations.

Screening frequency among women aged 75 years and older did not change significantly during our observation period. As with the other two age groups, the majority of women aged 75 years and older were annual screeners, with the proportion of nonregular screeners falling between those of women aged 40–49 and women aged 50–74. 

Regardless of age, our data support the benefits of annual screening mammography for early detection of breast cancer. With routine screening mammography, prior studies have shown that the three major prognostic features of breast cancer (tumor size, grade, and lymph node status) are significantly improved [11]. Additionally, stage at diagnosis is a well-known predictor of survival, with 99% survival for localized breast cancer compared to 84% in patients with regional disease and 23% for patients with distant disease [12]. We show that, when compared to biennial and nonregular screeners, regular screeners were more likely to present with early breast cancer, including carcinoma in situ (27%) and stage I (53%) invasive breast cancer. Additionally, over two-thirds of regular screeners presented with nonpalpable or clinically occult cancers, while about half of both biennial and nonregular screeners presented with palpable, later stage, and breast cancer.

 A retrospective analysis of the major screening trials found a strong association between risk of breast cancer mortality and diagnosis of advanced breast cancer. The approximately proportional decreased risk of advanced breast cancer and decrease in breast cancer mortality helped to show that screening leads to a decrease in the relative risk of advanced disease and subsequent mortality [13]. In a similar vein, Cady et al. noted that a reduced rate of advanced breast cancer can be used as a surrogate for success of screening, since screening mammography reduces mortality by earlier detection of biologically progressive cancers [5]. Moreover, a recent longitudinal prospective control cohort study found that woman aged 40–49 with mammographically detected cancers were not only more likely to have lower stage disease detection than those with patient or physician detected cancers but were also less likely to die of disease and less likely to have recurrences [14]. Therefore, though we are unable to assess the mortality benefit of screening in our population due to short-term followup, we are able to show success of screening by demonstrating earlier detection of clinically occult tumors in regular screeners as compared to more advanced, palpable tumors in less frequent and nonregular screeners.

The benefit of early detection of breast cancer afforded to annual screeners enables for a more conservative approach to treatment and allows surgical, medical, and radiation treatment to be more effective [14]. Breast conserving therapy (BCT), consisting of lumpectomy, possible node dissection, and adjuvant radiation, is a less morbid, more conservative treatment that is more likely to be an available option to regular screeners with early stage cancers as compared to more extensive treatments often required by the advanced cancers of nonregular screeners. Findings from a recent population-based prospective registry study support that BCT confers at least equivalent, if not superior, survival to mastectomy [15]. Additionally, detection of early stage breast cancer potentially spares the patient exposure to cytotoxic systemic chemotherapy, whose side effects are not just limited to the short term. In our population, in a speculative analysis, up to 48% of non-regular screeners could have potentially been spared chemotherapy had they undergone annual screening mammography. Notably, the harms of treating more advanced disease and increased recurrence risk in nonscreeners were not considered by the USPSTF when the guidelines were updated [16].

This study utilized a breast cancer database that prospectively enrolls all patients presenting to our institution, a high volume cancer center, for definitive surgical management of the breast cancer. Though the generalizability of data from a single, large urban academic institution can be questioned, our population of women represent a highly educated (65% with college degree or higher), insured population. As such, screening behavior in our population is unlikely to be confounded by access to mammography and instead more likely to be impacted by changes in guidelines and resulting recommendations of primary physicians. Another potential limitation of this study is that screening behavior was assessed by self-report via a questionnaire administered to patients on enrollment and is subject to recall bias. However, we were able to confirm the self-reported frequency with dates of mammograms prior to diagnosis and found 100% concordance. Lastly, since we started enrolling patients in January 2010, we cannot comment on screening behavior in our patient population prior to the updated USPSTF guidelines. Therefore, we are only able to evaluate the trend in screening in the years since the update and are unable to comment on how mammogram frequency changed as a result of these updates. While we can only speculate as to whether the impetus for this change is based on the patients' own decision making or due to changes in recommendations by primary physicians, it will be interesting to see if this trend continues as we continue to enroll more patients. 

## 5. Conclusion

Despite these limitations, our data are in line with prior studies that support the benefit of regular mammographic screening in detecting early breast cancer [5, 6]. We found that, in the years subsequent to the guideline updates, the overall screening frequency in our population did not change significantly. Our results suggest that women who are not screened annually are at increased risk of a delay in breast cancer diagnosis, which may impact treatment options and outcomes. 

## Figures and Tables

**Table 1:** Clinical characteristics between the years of 2010 and 2012.

Variables	Years and total *N* (%)				
	All years (*N* = 1112)	2010 (*N* = 431)	2011 (*N* = 430)	2012 (*N* = 251)	*P* value
Age group					
40–49 years	270 (24%)	102 (24%)	118 (27%)	50 (20%)	0.25
50–74 years	696 (63%)	271 (63%)	256 (60%)	169 (67%)	
75+ years	146 (13%)	58 (13%)	56 (13%)	32 (13%)	
Method of first detection					
Self-breast exam	272 (24%)	118 (28%)	97 (23%)	57 (23%)	0.40
Clinical breast exam	51 (5%)	23 (5%)	15 (3%)	13 (5%)	
Mammogram	684 (61%)	251 (58%)	270 (63%)	163 (65%)	
Ultrasound	53 (5%)	22 (5%)	21 (5%)	10 (4%)	
MRI	43 (4%)	14 (3%)	23 (5%)	6 (2%)	
Other	9 (1%)	3 (1%)	4 (1%)	2 (1%)	
Palpability					
Nonpalpable	787 (71%)	290 (67%)	318 (74%)	179 (71%)	0.10
Palpable	325 (29%)	141 (33%)	112 (26%)	72 (29%)	
Screening frequency					
Annual	772 (69%)	310 (72%)	295 (69%)	167 (67%)	0.30
Biennial	83 (8%)	36 (8%)	36 (8%)	11 (4%)	
Nonregular	193 (17%)	69 (16%)	78 (18%)	46 (18%)	
NA/missing	64 (6%)	16 (4%)	21 (5%)	27 (11%)	
Breast cancer stage					
Stage 0	260 (23%)	100 (23%)	109 (25%)	51 (20%)	0.66
Stage I	556 (50%)	212 (49%)	211 (49%)	133 (53%)	
Stage II	235 (21%)	97 (23%)	84 (20%)	54 (22%)	
Stage III	54 (5%)	19 (4%)	22 (5%)	13 (5%)	
Stage IV	1 (0.09%)	1 (0.23%)	0 (0%)	0 (0%)	
No residual cancer (after neoadjuvant)	6 (1%)	2 (1%)	4 (1%)	0 (0%)	
Histology					
DCIS	253 (23%)	99 (23%)	104 (24%)	50 (20%)	0.79
DCIS with microinvasion	21 (2%)	7 (2%)	11 (3%)	3 (1%)	
Invasive ductal carcinoma	663 (59%)	258 (60%)	250 (58%)	155 (62%)	
Invasive lobular carcinoma	108 (10%)	41 (9%)	40 (9%)	27 (11%)	
Mixed (tubular, papillary, medullary, etc.)	67 (6%)	26 (6%)	25 (6%)	16 (6%)	
ER status					
Negative < 10%	184 (17%)	59 (14%)	81 (19%)	44 (18%)	0.12
Positive ≥ 10%	912 (82%)	365 (85%)	343 (80%)	204 (81%)	
Unknown/missing	16 (1%)	7 (1%)	6 (1%)	3 (1%)	
PR status					
Negative < 10%	334 (30%)	118 (27%)	139 (32%)	77 (31%)	0.29
Positive ≥ 10%	762 (69%)	306 (71%)	285 (66%)	171 (68%)	
Unknown/missing	16 (1%)	7 (2%)	6 (2%)	3 (1%)	
HER-2 neu status					
Negative (0, 1+)	732 (66%)	279 (65%)	276 (64%)	177 (70%)	0.03
Positive (3+)	94 (8%)	36 (8%)	36 (8%)	22 (9%)	
Equivocal (2+)	12 (1%)	10 (2%)	2 (1%)	0 (0%)	
Unknown/missing	274 (25%)	106 (25%)	116 (27%)	52 (21%)	

MRI: magnetic resonance imaging; DCIS: ductal carcinoma in situ; ER: estrogen receptor; PR: progesterone receptor.

**Table 2:** Screening frequency by age groups over time.

Variables	Years and total *N* (%)				
40–49 years of age	All years (*N* = 256)	2010 (*N* = 98)	2011 (*N* = 114)	2012 (*N* = 44)	*P* value

Annual screening	155 (60%)	55 (56%)	72 (63%)	28 (64%)	0.45
Biennial screening	30 (12%)	16 (16%)	11 (10%)	3 (7%)	
Nonregular screening	71 (28%)	27 (28%)	31 (27%)	13 (29%)	

50–74 years of age	All years (*N* = 656)	2010 (*N* = 261)	2011 (*N* = 245)	2012 (*N* = 150)	*P* value

Annual screening	516 (79%)	212 (81%)	187 (76%)	117 (78%)	0.02
Biennial screening	42 (6%)	17 (7%)	22 (9%)	3 (2%)	
Nonregular screening	98 (15%)	32 (12%)	36 (15%)	30 (20%)	

75+ years of age	All Years (*N* = 136)	2010 (*N* = 56)	2011 (*N* = 50)	2012 (*N* = 30)	*P* value

Annual screening	101 (74%)	43 (77%)	36 (72%)	22 (73%)	0.32
Biennial screening	11 (8%)	3 (5%)	3 (6%)	5 (17%)	
Nonregular screening	24 (18%)	10 (18%)	11 (22%)	3 (10%)	

**Table 3:** Breast cancer stage at diagnosis and screening frequency.

Breast cancer	Screening frequency and total *N* (%)			*P* value
	Annual (*N* = 772)	Biennial (*N* = 83)	Nonregular (*N* = 193)	
Stage 0	206 (27%)	13 (16%)	28 (15%)	<0.0001
Stage 1	408 (53%)	36 (43%)	78 (40%)	
Stage 2	131 (17%)	29 (35%)	63 (33%)	
Stage 3	26 (3%)	2 (3%)	21 (11%)	
Stage 4	0 (0%)	1 (1%)	0 (0%)	

**Table 4:** Tumor palpability and screening frequency.

Palpability	Screening frequency and total *N* (%)			*P* value
	Annual (*N* = 772)	Biennial (*N* = 83)	Nonregular (*N* = 193)	
Nonpalpable	609 (79%)	45 (54%)	84 (44%)	<0.0001
Palpable	163 (21%)	38 (46%)	109 (56%)	
